# The Office of Iron in the Blood

**Published:** 1890-03

**Authors:** 


### THE OFFICE OF IRON IN THE BLOOD.

Iron exists in the blood in the red corpuscles, and gives them color and the power of absorbing gases. The fact that peroxide of iron is one of the readiest absorbents of gasses, and parts with them as readily on exposure in thin layers to the air, so that it can be used over and over again for that work, gives a clew to its special function in the red corpuscles of the blood. It enables them readily to absorb oxygen as they pass along the minute blood-vessels of the lungs and to carry it to all parts of the body, where they part with it as it is demanded. It is supposed, also, to take up carbonic acid in exchange for the oxygen it yields up, and to convey to the lungs that portion of this substance which is expired.

